# Development of Next-Generation Peptide Binders Using *In vitro* Display Technologies and Their Potential Applications

**DOI:** 10.3389/fimmu.2013.00224

**Published:** 2013-08-01

**Authors:** Akira Wada

**Affiliations:** ^1^RIKEN, Saitama, Japan

**Keywords:** peptide binder, *in vitro* display technology, target binding, peptide therapeutic, antibody drug

## Abstract

During the last decade, a variety of monoclonal antibodies have been developed and used as molecular targeting drugs in medical therapies. Although antibody drugs tend to have intense pharmacological activities and negligible side effects, several issues in their development and prescription remain to be resolved. Synthetic peptides with affinities and specificities for a desired target have received significant attention as alternatives to antibodies. *In vitro* display technologies are powerful methods for the selection of such peptides from combinatorial peptide libraries. Various types of peptide binders are being selected with such technologies for use in a wide range of fields from bioscience to medicine. This mini review article focuses on the current state of *in vitro* display selection of synthetic peptide binders and compares the selected peptides with natural peptides/proteins to provide a better understanding of the target affinities and inhibitory activities derived from their amino acid sequences and structural frameworks. The potential of synthetic peptide binders as alternatives to antibody drugs in therapeutic applications is also reviewed.

## Introduction

Since the 1990s, monoclonal antibodies have been developed as molecular targeting drugs to treat diseases such as cancers and inflammatory disorders (1, 2). More than 20 antibody drugs (e.g., Herceptin for breast cancer and Remicade for rheumatoid arthritis) have been launched to date and are considered perfect agents with intense pharmacological activities and no side effects. However, unavoidable issues have been revealed in their development and prescription (1, 2). For example, chimera antibody drugs that include part of a mouse antibody are likely to be eliminated by the human immune system and cause side effects through antibody-dependent cellular cytotoxicity. Additionally, various patents for antibody humanization introduce difficulty in some territories for the production of new antibody drugs. Therefore, the development of synthetic peptide binders with affinities for a desired target, particularly those in which peptides are the natural ligands, should provide an innovative solution to these problems. This mini review focuses on current studies related to *in vitro* display technologies for the selection of synthetic peptide binders and their use in biological, biotechnological, and medical studies. This also compares the features of these synthetic peptides with those of natural peptides/proteins to provide an understanding of the relationships among amino acid sequence, structural conformation, and affinity for a desired target that allow the prediction of their potential as alternatives to antibody reagents and drugs.

## Using Phage Display Technologies to Select Peptide Binders

Many recent studies have been undertaken to develop *in vitro* display technologies for the selection of peptide binders from combinatorial peptide libraries (CPLs). These technologies enable the creation of new peptides that can bind specifically to a wide range of target molecules [e.g., receptors, enzymes, viruses, materials, and small molecules (3–10)]. In particular, such peptides can be synthesized rapidly and precisely via automated chemical reactions and modified chemically to expand their functions and structures. Furthermore, in contrast to antibodies, synthetic peptides can be stored for long periods in both a solid state and solution, which facilitates large-scale production at reasonable costs. Owing to these advantages, synthetic peptide binders have attracted much attention over the years as alternatives to antibody reagents and drugs.

**Figure 1:**
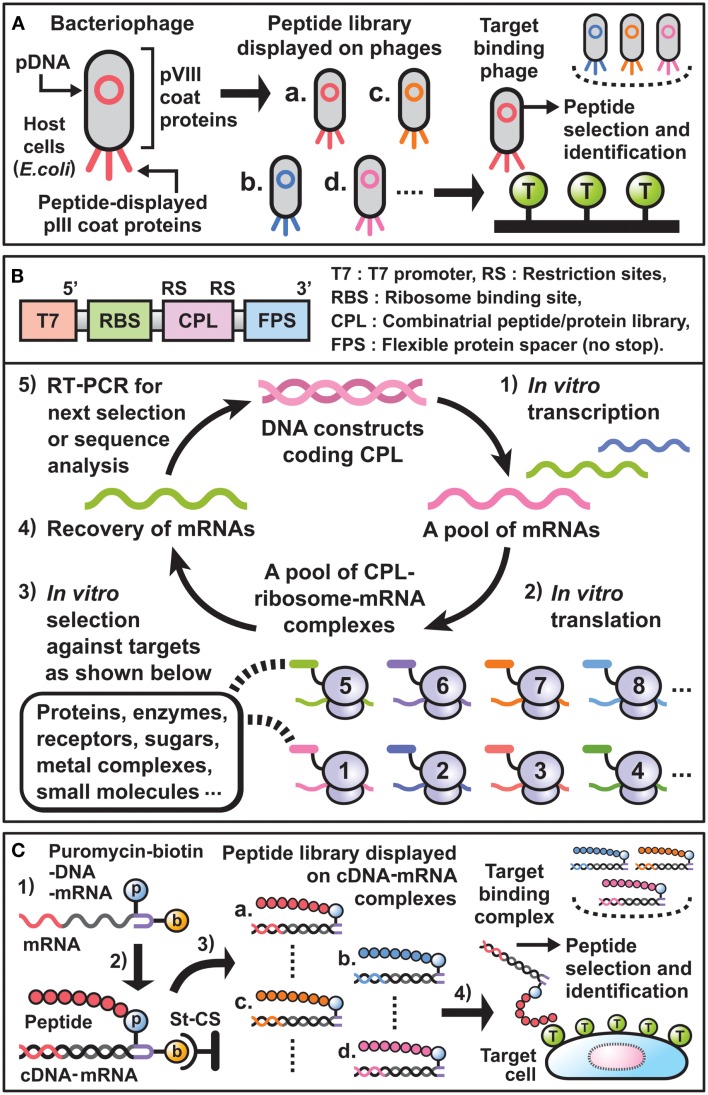
**Essential components and schemes for *in vitro* display selection**. **(A)** Scheme of phage display selection of peptide binders. Bacteriophages linking combinatorial peptides as phenotype with their plasmid DNAs (pDNAs) as genotype are produced in *E. coi*. A library of peptides displayed on the pIII coat proteins is panned against target-coated substrates. Then, target binding phages are selected and their pDNA sequences are analyzed to identify desirable peptide binders. **(B)** Upper panel: DNA constructs used for *E. coli* ribosome display technology. T7 promoter and RBS are necessary for *in vitro* transcription and translation, respectively. The coding sequences for CPL are inserted between suitable restriction sites and are followed by the coding sequence for FPS. Lower panel: cycle scheme of *E. coli* ribosome display selection of peptide binders. (1) DNA constructs are transcribed *in vitro* by T7 RNA polymerase to synthesize mRNAs. (2) The resulting mRNA pool is translated *in vitro* by a cell-free protein synthesis system extracted from *E. coli* to generate a library of ternary complex that contains CPL, ribosome, and mRNA. Since each mRNA encodes the sequence of the CPL fused to FPS without stop codons, which result in stalling of the ribosomes on mRNA, CPL–ribosome–mRNA complexes can be formed. (3) After mixing the complexes and target-coated beads/plates, desirable complexes that display peptides bound to target molecules are selected. (4) The mRNAs are recovered by dissociation of the selected complexes through the addition of EDTA. (5) The purified mRNAs are reverse-transcribed *in vitro* to synthesize cDNAs, and subsequently amplified by PCR. These resulting DNA constructs are directly used for the next selection cycle, or are analyzed by cloning and sequencing to identify new peptide binders. **(C)** Scheme of cDNA display selection of peptide binders. (1) The puromycin (p) and biotin (b) attached DNA linker is ligated with the 3′ end of mRNA encoding combinatorial peptide. (2) After immobilization of the construct through binding of biotin to streptavidin-coated surfaces (St-CS), *in vitro* synthesized peptide binds to the puromycin and the mRNA is subsequently transcribed to generate a peptide–cDNA–mRNA complex. (3) Digestion of restriction sites in the DNA linkers affords a library consisting of peptide–cDNA–mRNA complexes. (4) Through screen of the library against cells of interest, target binding complexes are recovered and their cDNA sequences are analyzed to identify desirable peptide binders.

During the last decade, phage display has been widely used for the selection of peptide binders or affinity maturation of antibodies (11–14). By following the scheme (Figure 1A), it becomes possible to discover unique peptides that dock to the sites of small molecule-protein interaction, the interfaces of protein–protein interaction, or the cavities for substrate-enzyme interaction. In particular, if the binding sites of newly selected peptides on a target molecule are nearly identical to those of natural ligands, they can be considered structural or functional mimics. For example, synthetic peptide binders as mimics of the small molecule “biotin,” which binds strongly to the tetrameric protein “streptavidin” found in *Streptomyces avidinii*, have been selected using phage display and are well characterized in terms of structural conformation and target-binding activity at the amino acid sequence level.

The first phage-displayed peptide mimics of biotin were discovered in a 15-mer peptide library of 10^7^, and their amino acid sequences commonly contain the His-Pro-Gln (HPQ) motif (15). In another study, this motif was also found in sequences of selected peptide binders against streptavidin (16). Furthermore, X-ray crystal structures have revealed that the HPQ motif is located at the ends of the β-barrels in streptavidin, and that the side chains of the motif interact with the biotin-binding site (17). Although the HPQ sequence is indispensable for streptavidin binding affinity and specificity, the dissociation constant, *K*_D_, of the peptide binders was calculated to be in the millimolar range. The affinities of the binders to streptavidin were very low compared to that of biotin (*K*_D_ = ∼10^−15^ M). Therefore, to stabilize the structures of the peptide binders and increase their affinities, libraries of disulfide-constrained cyclic peptides (CX_4_C, CX_5_C, CX_6_C; C, cysteine; X: 20 natural amino acids) were constructed and screened by phage display (18). The selected cyclic peptide binders had the HPQ motif and exhibited higher affinities, with *K*_D_ values in the nanomolar range, than those of the corresponding linear molecules. In addition, the X-ray crystal structure of a cyclic peptide bound to streptavidin showed that the interaction of the HPQ motif with streptavidin is identical to that of the linear peptide (19). These data demonstrate that the cyclic conformation resulting from disulfide bonding increases structural rigidity to decrease both solvent and peptide entropy, enhancing target affinity.

In a refinement of the cyclization concept, a library of bicyclic peptides has been synthesized through intramolecular cross-linking of tris-(bromomethyl)benzene with three cysteine residues in combinatorial peptide sequences (CX_6_CX_6_C) (20). The structurally constrained peptide library was screened against human plasma kallikrein by using a modified phage display. It is worth noting that the selected bicyclic peptide exhibited high inhibitory activity with an inhibition constant (*K*_i_) of 1.5 nM, and efficiently interrupted the intrinsic coagulation pathway in human plasma *ex vivo*. Moreover, various chemical approaches for synthesizing structurally constrained peptides are reported to strengthen target affinities and provide adequate function (21–23). These results demonstrate that structurally constrained peptides have both increased target affinity and proteolytic stability and also suggest that they are potential new drugs with the advantages of small molecules and biologics.

Many types of peptide binders have been successfully selected and used for multiple purposes. However, phage display-derived peptide binders have often had insufficient affinities against target molecules because phage display selection has several problems related to the use of living cells and bacteriophages themselves (e.g., steric repulsion between target molecules and phage coat proteins, cell toxicity of peptides, and the use of peptide libraries of small sizes of 10^7∼9^) (11, 12). These unavoidable phenomena tend to exclude desired peptide binders from CPLs through selection. Therefore, to circumvent these limitations and successfully select peptide binders adapted for wide-ranging uses, it has been necessary to develop improved display technologies that do not use living cells.

## Emergence of Cell-Free Display Technologies for the Selection of Peptide Binders

Against the above backdrop, ribosome display, mRNA display, and CIS display have been developed as *in vitro*, cell free, display technologies in combination with cell-free protein synthesis systems (24–26). In selecting peptide binders using these technologies, the preparation and use of transition complexes that link combinatorial peptides (phenotype) and the corresponding mRNAs or DNAs (genotype) is indispensable. For instance, *E. coli* ribosome display selection of peptide binders is generally carried out following the scheme (Figure 1B). Thus, new peptide binders with affinities and specificities for a required target can be selected from various peptide libraries in a small test tube.

Indeed, ribosome (polysome) display has enabled the selection of decapeptides with high affinities to monoclonal antibody from a pool of peptide–ribosome–mRNA complexes prepared from a 10^12^-member DNA library (27). Interestingly, most of the selected peptides contain a consensus sequence similar to that of the known epitope for the target antibody. Moreover, in ribosome display selection using a 15-mer peptide library of the large size of 10^13^ against streptavidin (28), the HPQ sequence essential for streptavidin binding was found in members of the selected peptides. Furthermore, the peptides and shortened variants possessed remarkably higher affinities, with *K*_D_ values in the low nanomolar range, than those of phage display-derived peptides. In another case, by screening a library of ribosomal complex stabilized by automatic association of a protein with an RNA motif at the 5′ terminus of the mRNA, disulfide-constrained peptides with metal binding affinities were successfully identified even at ambient temperature (29). Moreover, ribosome display can be optimized to screen not only peptide libraries but also single-chain fragment variable (scFv) and randomized protein libraries. In fact, *in vitro* selection of scFvs and their affinity maturation (30, 31), and exploration of structural proteins with affinities to desired targets (32, 33) have been carried out using improved ribosome display. Ribosome display is currently performed with various cell-free protein synthesis systems derived from eukaryotic cell extracts (34, 35) and reconstituted completely from the essential elements of 44 *E. coli* (36). Therefore, the ribosome display selection strategy is being generalized and applied in a wide range of research and development fields.

mRNA display selection of peptide binders can be executed using a scheme similar to that in Figure 1B. However, to link combinatorial peptides (phenotype) and their mRNAs (genotype), covalently linked peptide–mRNA complexes are used for selection (25). The covalent linkage in the complex is synthesized via reaction of the C-terminus of a nascent polypeptide with puromycin in the A-site of the ribosome through the formation of the corresponding peptide–ribosome–mRNA–puromycin complex. In contrast to ribosome display using peptide–ribosome–mRNA complexes, despite the time and effort required to synthesize puromycin-attached mRNAs and validate a purified library of peptide–mRNA complex, the covalent bonding between combinatorial peptides and their mRNAs is expected to enable successful selection of desirable peptide binders from large-size libraries (10^12 ∼ 14^) (37).

For example, in mRNA display selection against calmodulin (CaM) (38), previously known and novel CaM-binding proteins with various affinities were identified from a natural protein library. More than 2000 peptide binders were isolated from a combinatorial peptide library of ∼10^12^, some of which had CaM-binding motifs found in natural proteins. In addition, the selected peptides bound to CaM tightly, with unique secondary structures that differed from the conventional ones. These results imply that *in vitro* peptide/protein selection strategy has the potential to discover unknown protein-peptide/-protein interactions in various cell-signaling pathways (39, 40). Moreover, the high stability of the covalently linked peptide–mRNA complexes under physiological conditions allows their chemical modification through the reaction of functional groups of peptides with organic compounds. For instance, a hybrid drug-peptide library has been constructed via reaction of a cysteine side chain in the combinatorial peptide sequence (X_5_CX_5_) with 6-bromoacetyl penicillinate (41). Subsequently, the library (∼10^12^) of synthetic peptides, each bearing a pendant penicillin moiety, was screened against *Staphylococcus aureus* penicillin-binding protein 2A (PBP2A). This selection yielded a novel hybrid penicillin-peptide binder with 100-fold higher activity than that of penicillin itself. The results indicate that this approach might be a convenient way to increase the efficiency of current drugs and create powerful hybrid ligands. Furthermore, to produce unique peptide inhibitors with proteolytic stability, an mRNA display library of cyclic peptides was synthesized by intramolecular cross-linking of disuccinimidyl glutarate with two primary amines, those of methionine at the N-terminus and a lysine fixed in the combinatorial peptide sequence (MX_10_K; M: methionine, K: lysine) (42). The resulting library was screened against Gαi1, which is related to G-protein-coupled receptor (GPCR) signaling. The identified cyclic peptide binder exhibited strong affinity with *K*_D_ of ∼2.1 nM, similar to those of monoclonal antibodies and higher than that of an endogenous Gαi1 ligand. Additionally, the cyclic structure of the selected peptide enhanced its resistance to protease degradation compared with that of the parent linear peptide. Thus, cyclization in the internal peptide sequence is a useful approach for improving proteolytic stability and affinity for a desired target.

Currently, a cell-free protein synthesis system can be completely reconstituted with recombinant elements and purified ribosomes from *E. coli* (43) and used to produce proteins/peptides consisting of not only natural but also unnatural amino acids in combination with chemically modified amino acyl-tRNAs (44, 45). Reconstituted ribosomal translational machinery has been used in attempts to incorporate unnatural amino acids with various functional groups or backbone structures into combinatorial peptides to generate unnatural peptide–mRNA libraries. For example, a library of unnatural cyclic peptides has been constructed through random incorporation of 12 unnatural amino acids and the reaction of a dibromoxylene cross-linker with two cysteine residues fixed in the combinatorial peptide sequence (C(U/X)_10_C; U, unnatural amino acids) (46). Subsequently, mRNA display selection using a library of 10^13^ highly modified peptides was carried out against thrombin as a target molecule. Interestingly, the identified cyclic peptides possessed unnatural amino acids essential for binding to the target, and their affinities (*K*_D_ in the low nanomolar range) were much higher than those of their linear counterparts. Furthermore, their high inhibitory activities indicated that unnatural cyclic peptide binders are a potential new class of drug-like molecules.

Naturally occurring peptides are known to use the cyclic structure and *N*-methylated backbone for structural rigidity, specific target affinity, and proteolytic stability. Synthetic peptides and mimics with natural peptide-like structures are likely drug-like molecules with high affinity and specificity against targets *in vitro* and *in vivo* (47–49). Based on this concept, a cell-free protein synthesis system coupled with various chemically modified amino acyl-tRNAs has been developed to produce combinatorial peptides consisting of natural and *N*-methyl amino acids (*N*m) (50). Furthermore, chloroacetylated d-amino acid (ClAc) was effectively introduced at the N-terminus using this system to facilitate internal cyclization via the reaction of ClAc with a fixed cysteine in the peptide sequence. Subsequently, a 10^12^-member mRNA display library of *N*-methyl cyclic peptides [ClAc(*N*m/X)_8–15_C] was constructed through *in vitro* translation driven by the reconstituted system and was screened against the E6AP HECT domain (51). Remarkably, an isolated *N*-methyl cyclic peptide bound strongly to the target with a single-digit nanomolar *K*_D_ and inhibited the E6AP catalysis of polyubiquitination of proteins such as p53. In addition, cyclization of all characterized peptides enhanced their target affinities compared with those of the corresponding linear peptides. The successful identification and characterization of natural-like peptide inhibitors indicate that mRNA display selection would be useful for generating drug-like peptides that can bridge the gap between small molecule drugs and biologics.

## Prospects for Next-Generation Peptide Binders as Molecular Target Drugs

Previous studies have revealed that *in vitro* display selection of peptides from a wide variety of libraries is a powerful approach for the development of synthetic peptide binders against target molecules. Indeed, various peptide binders have been successfully selected and used in biology, biotechnology, and biomedical science. Furthermore, characterization of the selected peptides suggests that synthetic peptide binders might have *in vitro* molecular targeting activities comparable to those of antibodies. Based on these prospects, modified mRNA display [cDNA display (52)] selection methods have been developed to produce new peptides that can bind specifically to GPCRs as major drug targets or to cancer cells themselves *in vivo* (Figure 1C).

For instance, as shown in Figure 1C, a library of combinatorial peptide (X_8_)–cDNA–mRNA complexes has been constructed (53) and directly screened against growth hormone secretagogue receptors (GHSRs) expressed on CHO cells in culture medium. The selected peptides exerted an antagonistic effect on GHSR to suppress the contraction of isolated animal stomach induced by ghrelin as a natural ligand. In addition, intravenous administration of these peptides inhibited food intake in mice. Although the observed inhibitory activity was not as high as that of present agents, these data contribute to the future development of anti-obesity peptide drugs.

Conversely, a library of combinatorial peptide (X_15_)–cDNA–mRNA complexes has been screened against mammalian cancer cells under culture conditions (54). Subsequently, a group of peptides binding to cells was collected and re-screened according to the membrane permeability in a wide variety of cancer cell lines (HeLa, Lovo, A549, MCF-7, MKN45, HepG2, LNCap, KPK, U2OS, RC-15, RD-ES, H28, K562, and U251). Some of the final selected peptides could penetrate only into specific types of primary cancer cells of colon adenocarcinoma or acute myelogenous leukemia (AML). One of these cell-penetrating peptides was fused with the minimal p16 inhibitory sequence in a type of retro-inverso peptide (55) to induce the apoptosis of target cancer cells *in vivo*. Administration of the fusion peptide to NOD-SCID mice treated with AML cells or xenografts specifically inhibited tumor metastasis and growth. Therefore, these studies indicate that cell-based selection using *in vitro* display technologies have the potential to discover unprecedented peptide binders with molecular targeting and inhibitory abilities *in vivo*.

In conclusion, this review highlights the utility and versatility of *in vitro* display technologies for the selection of peptide binders suitable for multiple purposes in biology, biotechnology, and medical science. Furthermore, characterization of the selected peptides promises that synthetic peptide binders have potential as molecular target drugs comparable to that of monoclonal antibodies. However, to develop peptide-derived drugs that can be applied in a general medical setting, several issues (potential immunogenicity, protease degradation, and poor metabolic stability) remain to be resolved for human administration. Therefore, establishing an innovative strategy including *in vitro* display selection is necessary to discover peptide binders, make chemical modifications to provide protease resistance and metabolic stability, obtain structural information to optimize target-binding modes, and perform theoretical simulation to predict dynamic actions. Such an integrated strategy would facilitate the development of next-generation peptide binders and mimics as alternatives to monoclonal antibodies and native ligands, respectively. The emergence of peptide binders that overcome the above difficulties will allow us to tailor peptide drugs for the prevention, diagnosis, and treatment of a wide variety of diseases and cancers.

## Conflict of Interest Statement

The author declares that the research was conducted in the absence of any commercial or financial relationships that could be construed as a potential conflict of interest.

## References

[1] Clark M Antibody humanization: a case of the “Emperor’s new clothes?” Immunol Today (2000) 21:397–402 10.1016/S0167-5699(00)01680-7 10916143

[2] Presta LG Molecular engineering and design of therapeutic antibodies. Curr Opin Immunol (2008) 20:460–70 10.1016/j.coi.2008.06.012 18656541

[3] Cwirla SE Balasubramanian P Duffin DJ Wagstrom CR Gates CM Singer SC Peptide agonist of the thrombopoietin receptor as potent as the natural cytokine. Science (1997) 276:1696–9 10.1126/science.276.5319.1696 9180079

[4] Su JL Lai KP Chen CA Yang CY Chen PS Chang CC A novel peptide specifically binding to interleukin-6 receptor (gp80) inhibits angiogenesis and tumor growth. Cancer Res (2005) 65:4827–35 10.1158/0008-5472.CAN-05-0188 15930303

[5] Hyde-DeRuyscher R Paige LA Christensen DJ Hyde-DeRuyscher N Lim A Fredericks ZL Detection of small-molecule enzyme inhibitors with peptides isolated from phage-displayed combinatorial peptide libraries. Chem Biol (2000) 7:17–25 10.1016/S1074-5521(00)00062-4 10662687

[6] Welch BD VanDemark AP Heroux A Hill CP Kay MS Potent D-peptide inhibitors of HIV-1 entry. Proc Natl Acad Sci U S A (2007) 104:16828–33 10.1073/pnas.0708109104 17942675 PMC2040420

[7] Matsubara T Onishi A Saito T Shimada A Inoue H Taki T Sialic acid-mimic peptides as hemagglutinin inhibitors for anti-influenza therapy. J Med Chem (2010) 53:4441–9 10.1021/jm1002183 20476787

[8] Whaley SR English DS Hu EL Barbara PF Belcher AM Selection of peptides with semiconductor binding specificity for directed nanocrystal assembly. Nature (2000) 405:665–8 10.1038/35015043 10864319

[9] Wang S Humphreys ES Chung SY Delduco DF Lustig SR Wang H Peptides with selective affinity for carbon nanotubes. Nat Mater (2003) 2:196–200 10.1038/nmat833 12612679

[10] Rodi DJ Janes RW Sanganee HJ Holton RA Wallace BA Makowski L Screening of a library of phage-displayed peptides identifies human bcl-2 as a taxol-binding protein. J Mol Biol (1999) 285:197–203 10.1006/jmbi.1998.2303 9878399

[11] Smith GP Petrenko VA Phage display. Chem Rev (1997) 97:391–410 10.1021/cr960065d 11848876

[12] Hoess RH Protein design and phage display. Chem Rev (2001) 101:3205–18 10.1021/cr000056b 11710069

[13] Eisenhardt SU Schwarz M Bassler N Peter K Subtractive single-chain antibody (scFv) phage-display: tailoring phage-display for high specificity against function-specific conformations of cell membrane molecules. Nat Protoc (2007) 2:3063–73 10.1038/nprot.2007.455 18079705

[14] Bradbury AR Sidhu S Dübel S McCafferty J Beyond natural antibodies: the power of *in vitro* display technologies. Nat Biotechnol (2011) 29:245–54 10.1038/nbt.1791 21390033 PMC3057417

[15] Devlin JJ Panganiban LC Devlin PE Random peptide libraries: a source of specific protein binding molecules. Science (1990) 249:404–6 10.1126/science.2143033 2143033

[16] Lam KS Salmon SE Hersh EM Hruby VJ Kazmierski WM Knapp RJ A new type of synthetic peptide library for identifying ligand-binding activity. Nature (1991) 354:82–4 10.1038/354082a0 1944576

[17] Weber PC Pantoliano MW Thompson LD Crystal structure and ligand-binding studies of a screened peptide complexed with streptavidin. Biochemistry (1992) 31:9350–4 10.1021/bi00154a004 1390720

[18] Giebel LB Cass RT Milligan DL Young DC Arze R Johnson CR Screening of cyclic peptide phage libraries identifies ligands that bind streptavidin with high affinities. Biochemistry (1995) 34:15430–5 10.1021/bi00047a006 7492543

[19] Katz BA Binding to protein targets of peptidic leads discovered by phage display: crystal structures of streptavidin-bound linear and cyclic peptide ligands containing the HPQ sequence. Biochemistry (1995) 34:15421–9 10.1021/bi00047a005 7492542

[20] Heinis C Rutherford T Freund S Winter G Phage-encoded combinatorial chemical libraries based on bicyclic peptides. Nat Chem Biol (2009) 5:502–7 10.1038/nchembio.184 19483697

[21] Henchey LK Jochim AL Arora PS Contemporary strategies for the stabilization of peptides in the alpha-helical conformation. Curr Opin Chem Biol (2008) 12:692–7 10.1016/j.cbpa.2008.08.019 18793750 PMC2650020

[22] Timmerman P Shochat SG Desmet J Barderas R Casal JI Meloen RH Binding of CDR-derived peptides is mechanistically different from that of high-affinity parental antibodies. J Mol Recognit (2010) 23:59–68 10.1002/jmr.1017 21038356

[23] Smeenk LE Dailly N Hiemstra H van Maarseveen JH Timmerman P Synthesis of water-soluble scaffolds for peptide cyclization, labeling, and ligation. Org Lett (2012) 14:1194–7 10.1021/ol203259a 22332901

[24] Zahnd C Amstutz P Plückthun A Ribosome display: selecting and evolving proteins *in vitro* that specifically bind to a target. Nat Methods (2007) 4:269–79 10.1038/nmeth1003 17327848

[25] Cotton SW Zou J Valencia CA Liu R Selection of proteins with desired properties from natural proteome libraries using mRNA display. Nat Protoc (2011) 6:1163–82 10.1038/nprot.2011.354 21799486

[26] Odegrip R Coomber D Eldridge B Hederer R Kuhlman PA Ullman C CIS display: *in vitro* selection of peptides from libraries of protein-DNA complexes. Proc Natl Acad Sci U S A (2004) 101:2806–10 10.1073/pnas.0400219101 14981246 PMC365701

[27] Mattheakis LC Bhatt RR Dower WJ An *in vitro* polysome display system for identifying ligands from very large peptide libraries. Proc Natl Acad Sci U S A (1994) 91:9022–6 10.1073/pnas.91.19.9022 7522328 PMC44739

[28] Lamla T Erdmann VA Searching sequence space for high-affinity binding peptides using ribosome display. J Mol Biol (2003) 329:381–8 10.1016/S0022-2836(03)00432-7 12758084

[29] Wada A Sawata SY Ito Y Ribosome display selection of a metal-binding motif from an artificial peptide library. Biotechnol Bioeng (2008) 101:1102–7 10.1002/bit.21975 18613123

[30] Hanes J Schaffitzel C Knappik A Plückthun A Picomolar affinity antibodies from a fully synthetic naive library selected and evolved by ribosome display. Nat Biotechnol (2000) 18:1287–92 10.1038/82407 11101809

[31] Luginbühl B Kanyo Z Jones RM Fletterick RJ Prusiner SB Cohen FE Directed evolution of an anti-prion protein scFv fragment to an affinity of 1 pM and its structural interpretation. J Mol Biol (2006) 363:75–97 10.1016/j.jmb.2006.07.027 16962610

[32] Binz HK Amstutz P Plückthun A Engineering novel binding proteins from nonimmunoglobulin domains. Nat Biotechnol (2005) 23:1257–68 10.1038/nbt1127 16211069

[33] Tamaskovic R Simon M Stefan N Schwill M Plückthun A Designed ankyrin repeat proteins (DARPins) from research to therapy. Methods Enzymol (2012) 503:101–34 10.1016/B978-0-12-396962-0.00005-7 22230567

[34] He M Taussig MJ Eukaryotic ribosome display with in situ DNA recovery. Nat Methods (2007) 4:281–8 10.1038/nmeth0907-763 17327849

[35] Douthwaite JA Eukaryotic ribosome display selection using rabbit reticulocyte lysate. Methods Mol Biol (2012) 805:45–57 10.1007/978-1-61779-379-0_3 22094799

[36] Ueda T Kanamori T Ohashi H Ribosome display with the PURE technology. Methods Mol Biol (2010) 607:219–25 10.1007/978-1-60327-331-2_18 20204860

[37] Cho G Keefe AD Liu R Wilson DS Szostak JW Constructing high complexity synthetic libraries of long ORFs using in vitro selection. J Mol Biol (2000) 297:309–19 10.1006/jmbi.2000.3571 10715203

[38] Huang BC Liu R Comparison of mRNA-display-based selections using synthetic peptide and natural protein libraries. Biochemistry (2007) 46:10102–12 10.1021/bi700220x 17685586

[39] Horisawa K Tateyama S Ishizaka M Matsumura N Takashima H Miyamoto-Sato E *In vitro* selection of Jun-associated proteins using mRNA display. Nucleic Acids Res (2004) 32:e169 10.1093/nar/gnh167 15576676 PMC535696

[40] Fujimori S Hirai N Ohashi H Masuoka K Nishikimi A Fukui Y Next-generation sequencing coupled with a cell-free display technology for high-throughput production of reliable interactome data. Sci Rep (2012) 2:691 10.1038/srep00691 23056904 PMC3466446

[41] Li S Roberts RW A novel strategy for in vitro selection of peptide-drug conjugates. Chem Biol (2003) 10:233–9 10.1016/S1074-5521(03)00047-4 12670537

[42] Millward SW Fiacco S Austin RJ Roberts RW Design of cyclic peptides that bind protein surfaces with antibody-like affinity. ACS Chem Biol (2007) 2:625–34 10.1021/cb7001126 17894440 PMC3747972

[43] Shimizu Y Inoue A Tomaril Y Suzuki T Yokogawa T Nishikawa K Cell-free translation reconstituted with purified components. Nat Biotechnol (2001) 19:s751–5 10.1038/90802 11479568

[44] Josephson K Hartman MC Szostak JW Ribosomal synthesis of unnatural peptides. J Am Chem Soc (2005) 127:11727–35 10.1021/ja0515809 16104750

[45] Kajihara D Abe R Iijima I Komiyama C Sisido M Hohsaka T FRET analysis of protein conformational change through position-specific incorporation of fluorescent amino acids. Nat Methods (2006) 3:923–9 10.1038/nmeth945 17060916

[46] Schlippe YV Hartman MC Josephson K Szostak JW *In vitro* selection of highly modified cyclic peptides that act as tight binding inhibitors. J Am Chem Soc (2012) 134:10469–77 10.1021/ja301017y 22428867 PMC3384292

[47] Patch JA Barron AE Mimicry of bioactive peptides via non-natural, sequence-specific peptidomimetic oligomers. Curr Opin Chem Biol (2002) 6:872–7 10.1016/S1367-5931(02)00385-X 12470744

[48] Sit CS Yoganathan S Vederas JC Biosynthesis of aminovinyl-cysteine-containing peptides and its application in the production of potential drug candidates. Acc Chem Res (2011) 44:261–8 10.1021/ar1001395 21366289

[49] Baeriswyl V Heinis C Polycyclic peptide therapeutics. ChemMedChem (2013) 8:377–84 10.1002/cmdc.201200513 23355488

[50] Kawakami T Murakami H Suga H Messenger RNA-programmed incorporation of multiple N-methyl-amino acids into linear and cyclic peptides. Chem Biol (2008) 15:32–42 10.1016/j.chembiol.2007.12.008 18215771

[51] Yamagishi Y Shoji I Miyagawa S Kawakami T Katoh T Goto Y Natural product-like macrocyclic *N*-methyl-peptide inhibitors against a ubiquitin ligase uncovered from a ribosome-expressed de novo library. Chem Biol (2011) 18:1562–70 10.1016/j.chembiol.2011.09.013 22195558

[52] Mochizuki Y Biyani M Tsuji-Ueno S Suzuki M Nishigaki K Husimi Y One-pot preparation of mRNA/cDNA display by a novel and versatile puromycin-linker DNA. ACS Comb Sci (2011) 13:478–85 10.1021/co2000295 21766868

[53] Ueno S Yoshida S Mondal A Nishina K Koyama M Sakata I *In vitro* selection of a peptide antagonist of growth hormone secretagogue receptor using cDNA display. Proc Natl Acad Sci U S A (2012) 109:11121–6 10.1073/pnas.1203561109 22723348 PMC3396546

[54] Kondo E Saito K Tashiro Y Kamide K Uno S Furuya T Tumour lineage-homing cell-penetrating peptides as anticancer molecular delivery systems. Nat Commun (2012) 3:951 10.1038/ncomms1952 22805558

[55] Kondo E Tanaka T Miyake T Ichikawa T Hirai M Adachi M Potent synergy of dual antitumor peptides for growth suppression of human glioblastoma cell lines. Mol Cancer Ther (2008) 7:1461–71 10.1158/1535-7163.MCT-07-2010 18566217

